# Closing the Policy-Practice Gap in the Management of Child Contacts of Tuberculosis Cases in Developing Countries

**DOI:** 10.1371/journal.pmed.1001105

**Published:** 2011-10-11

**Authors:** Philip C. Hill, Merrin E. Rutherford, Rick Audas, Reinout van Crevel, Stephen M. Graham

**Affiliations:** 1 Centre for International Health, Department of Preventive and Social Medicine, University of Otago School of Medicine, Dunedin, New Zealand; 2 Department of Preventive and Social Medicine, University of Otago School of Medicine, Dunedin, New Zealand; 3 Department of Medicine, Radboud University Njimegen Medical Centre, Njimegen, The Netherlands; 4 Centre for International Child Health, Department of Paediatrics, University of Melbourne, Melbourne, Australia; 5 International Union Against Tuberculosis and Lung Disease, Paris, France

## Abstract

Philip Campbell Hill and colleagues propose using a health needs assessment framework, research tools, and a strategy for clinical evaluation to help better manage child contacts of adult TB cases.

**Summary Points**

- Children in contact with an adult with smear-positive tuberculosis (TB) are at high risk of being infected themselves and progressing to TB disease.
- The World Health Organization recommends that such children, if aged under 5 years, should receive preventive treatment once TB disease has been ruled out.
- This policy is rarely implemented and attempts to do so have had disappointing results.
- We propose a new approach using a health needs assessment framework, research tools, and a strategy for clinical evaluation.
- We show how this approach could be applied and evaluated by National TB Control Programs.

## The Policy-Practice Gap Regarding Children in Contact with a Tuberculosis Case

The prevention, diagnosis, and treatment of tuberculosis (TB) in children are of particular importance in developing countries where TB is endemic [1]. Child contacts of an adult with sputum smear–positive TB are at high risk of infection with *Mycobacterium tuberculosis* and subsequent early progression to TB disease [2]. Anti-tubercular antibiotic prophylaxis is highly effective in preventing progression to disease in children infected with *M. tuberculosis*, with protection of up to 90% [3]. Therefore, the World Health Organization (WHO) recommends that all children <5 years who are a household contact of a sputum smear–positive case should receive preventive treatment, once TB disease has been ruled out [4]. Box 1 summarises the 2006 WHO recommendation for contact management as a symptom-based approach, whereby most child contacts can be placed immediately on preventive treatment without the need for formal clinical evaluation.

**Box 1. A symptom-based approach to child contacts of adult TB cases**

Children who are household contacts of a sputum smear–positive adult TB case are initially evaluated in the community. If asymptomatic and less than 5 years of age, they are immediately commenced on preventive treatment. If they have symptoms consistent with TB disease they are referred for clinical workup. Those diagnosed with TB disease undergo a full course of multi-drug treatment. Those less than 5 years of age who are not diagnosed with TB disease receive preventive treatment.

*2006 WHO Guidance for National Tuberculosis Programmes on the Management of Tuberculosis in Children.*

Of concern, the WHO recommendation for the management of child contacts of a sputum smear–positive index case is rarely implemented, despite being incorporated widely into National TB Control Program (NTP) guidelines [5]–[7]. Possible reasons for this include that limited NTP resources are focused on the management of TB disease, the perceived need for specialised services and investigations to provide adequate clinical evaluation, and concerns regarding re-infection and poor adherence in relation to the development of resistance [7]–[10]. Furthermore, attempts to implement the policy have been characterised by low attendance for screening, poor adherence to preventive treatment, and high defaulting rates [9],[11],[12]. Specific barriers in relation to preventive treatment that have been identified include issues of knowledge, understanding, and perception in TB patients and TB program staff [5],[13], lack of an appropriate management structure and necessary tools [6], treatment side effects [14], transport difficulties, and cost [10],[13],[14]. However, the literature with respect to barriers in the management of child contacts of TB cases in developing countries is relatively sparse.

It is clear that there is a policy-practice gap that needs to be addressed. Here, we propose an evidence-based approach to close this gap and show how this can lead to the management of child contacts of TB cases being properly incorporated into NTP activities, applied in the community and clinic, and formally evaluated.

## Closing the Gap

**Figure 1:**
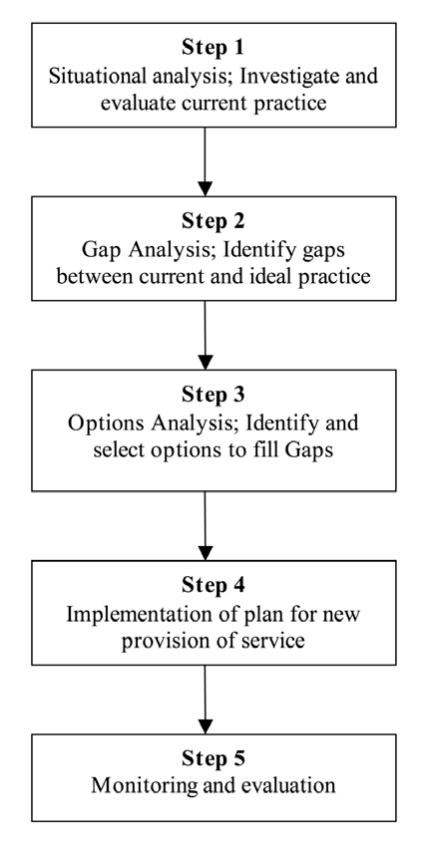
A health needs assessment framework for addressing the policy-practice gap in the management of child contacts of TB cases.

We propose the employment of an innovative combination of a health needs assessment (HNA) public health framework, integrated research tools, and a clinical evaluation plan in those child contacts with symptoms. The key steps of an HNA as a public health framework are described in Figure 1 [15]. An initial situational analysis of current practice is followed by identification of the gaps between current and ideal practice. This is followed by a process whereby options for filling the gaps are considered and the most appropriate recommended. These are then brought together and implemented as new routine practice. Once implemented, the new service is monitored and evaluated. This public health framework has been used to match health needs and provision in populations [15]. Quantitative and qualitative research tools have been integrated to provide a clear understanding of need [16]. The framework can be applied to specific health issues. For example, it has been adopted as a tool to develop a way forward for the rational use of oxygen in child pneumonia in West Africa [17],[18]. To the best of our knowledge, other than the step of situational analysis [5], it has not been applied to child TB case contact management.

### Situational Analysis

The indicators that should be included in the situational analysis of the management of child contacts of adult TB cases are summarised in Table 1. These cover basic demographic details of the TB cases that attend clinics and their child contacts, the performance and capacity of the system to deliver proper evaluation and management of TB contacts, key aspects of drug supply and quality, adherence and defaulting in relation to preventive and curative treatment, treatment outcome, attitudes and acceptability of child contact management by staff and primary caregivers, and cost analyses.

**Table 1:** Indicators of system performance in child TB case contact management.

Parameter	Indicator	Specific Study Details/Design
**Screening and diagnosis**	Number of child contacts of TB cases and their basic characteristics	Routinely record details of all cases and their household contacts
	Proportion of children who attend for screening in community and at the clinic	Cohort study of consecutive household contacts (*n* = 500)^a^
	Proportion of children diagnosed with or without TB that are misclassified	Review of all case notes over the previous 6 months
	Proportion of children who require clinical follow up to clarify diagnosis	Review of all case notes over the previous 6 months
	Proportion of those who require follow-up that complete it to a diagnostic decision	Review of all case notes over the previous 6 months
**Medication**	Availability and consistency of medicine supply	Annual survey of supply outlets
	Current quality	Annual analysis of drug quality in random samples
	Current cost	Annual review of cost
**Adherence/acceptability**	Proportion of adherent children	Cohort of consecutively treated children (*n* = 500)^b^
	Proportion of temporary default children	Cohort of consecutively treated children (*n* = 500)^b^
	Proportion of permanent default children	Cohort of consecutively treated children (*n* = 1,000)^b^
	Patient/parent acceptability	Qualitative survey of caregivers (*n* = 30-50)
	Clinician and staff acceptability	Qualitative survey of staff from various disciplines (*n* = 30–50)
**Treatment outcome**	Number of children (<5 years) on preventive treatment who develop TB	Cohort of consecutively treated children, 1 year follow-up (*n* = 2,000)^c^
	Proportion with side effects of preventive treatment	Cohort of consecutively treated children (*n* = 200)^d^
	Proportion stopping treatment because of side effects of medication	Cohort of consecutively treated children (*n* = 200)^d^
**Cost**	Cost to clinic	Survey of key staff (*n* = 10)
	Direct and indirect costs to child and caregiver	Survey of primary caregivers (*n* = 50)

a Index cases interviewed at diagnosis to identify case, contact, and household factors associated with non-attendance. A study of 500 contacts is advised, assuming non-attendance of at least 20%.

b Cohort study of 500 enables evaluation of risk factors for non-adherence (taking <80% of doses) and temporary defaulting (not taking medicine for at least one week), assuming at least 20% non-adherence; enlarged to 1,000 assuming permanent default rate is at least 10%.

c *n* = 2,000 is estimated to identify “secondary cases” on the basis of 60%–90% efficacy of preventive therapy, an assumption of >80% adherence and a natural progression off treatment of up to 20% over one year [2].

d Cohort of 200 is based on an expected incidence of symptomatic hepatotoxicity due to IPT of <10% over a treatment course in children [25].

The research tools that are required include relatively large cross-sectional and cohort studies as well as qualitative methods and focused cost questionnaires. We note that longitudinal follow-up of those placed on preventive treatment currently offers limited information at the situational analysis stage if preventive treatment is not practiced. We propose a single cohort study with recruitment extended to meet the requirements of each question (up to a maximum of 2,000 contacts for the disease outcome) and sufficient follow-up time of 1 year. Such an approach enables risk factors and barriers related to particular gaps to be identified (Table 2) and fed into the evidence base for the options process (Table 3). It is not absolutely necessary to have a secondary disease outcome indicator, as the efficacy of preventive therapy can be assumed from the literature. However, a baseline understanding of the incidence rate of secondary disease in a particular population is valuable for future comparisons. Cost analyses include both direct (costs to the patient/contacts and the health care provider to access care and provide treatment) and indirect costs (non-health care costs that result from engagement with the health system). By collecting direct and indirect cost information for exposed children who have preventive treatment and those who do not, the importance of economic barriers to screening and therapy can be established. For such an evaluation, an asset-based measure of wealth can be used to estimate the extent to which financial constraints impact on caregivers' decisions [19]. The qualitative studies provide new insights into factors that may be important with respect to attendance at screening and adherence to medication, as well as caregiver and staff knowledge and attitudes.

**Table 2:** Possible examples of identification of gaps with respect to IPT and possible relevant site-specific risk factors identified from the situational analysis.

Issue	Current Reality	Ideal	Gap	Site-Specific Factors Identified
**Screening and diagnosis**	Proportion attending screening 30%	Target >80% to attend screening^a^	>50% of expected case contacts are not screened	Knowledge in TB cases and staff, cost of attendance and travel time
	Proportion of children screened that are diagnosed with TB disease 50%	<10% are expected to have TB disease at screening [10]	>40% of children evaluated are inappropriately diagnosed with TB	Overuse and over-diagnosis of X-rays
**Adherence/acceptability**	40% adherence to preventive treatment	Target >80%^a^ adherence to preventive treatment	>40% excess of non-adherent children	Poor adherence, especially in the second half of treatment due to cumulative travel, time, and cost factors

a Targets for adherence to screening and treatment suggested here are broadly in line with WHO targets for the identification and management of tuberculosis cases.

**Table 3:** Examples of possible options analyses and recommendations to address gaps that are identified in the management of child contacts of adult TB cases.

Problem	Options	Evidence Analysis	Feasibility Analysis	Recommendation
**Low screening attendance due to knowledge gap, distance, and cost issues**	1. Educational video	Well received in relation to TB disease [26]	Subject to equipment and expertise availability	Develop and evaluate educational video for TB patients and contacts
	2. Decentralised symptom-based screening in the community and at community health centres	Decentralised provision of TB treatment improves uptake and completion [27]; symptom-based screening is safe [28]	Low cost; increased community clinic work load.	Symptom-based screening in the community; clinical evaluation and management at community clinics
	3. Cash transfer to stimulate attendance and subsequent adherence	Conditional cash transfer systems increase attendance at preventive treatment programmes [29]. Cost effectiveness is unclear.	Significant cost implications up front that need to be addressed	Pilot a cash transfer intervention with before and after evaluation, subject to finances
**Over-diagnosis of TB disease in children**	1. Specialised training in diagnosis of TB disease in children	Limited published evidence of effect on diagnostic accuracy	2 day centralised training courses are the most cost-effective	Introduce 2- to 5-day in-country training in diagnosis of child TB disease annually
	2. Remote interpretation of digital chest X-rays by WHO-accredited radiologists	Quality of X-rays and reading is acceptable and reliable	High cost of installation of digital X-ray machines	Consider digital X-ray and remote reading if high levels of over-diagnosis persist after training
**Low adherence**	1.Changing therapy from 6 months INH to 3 months RIF and INH	Efficacy is equivalent in adults but unclear if side effect profile is worse for 3-month regimen [30]. Some evidence of equivalent efficacy and improved adherence in children [8].	Lower cost; one extra medicine	Cohort study required for side effect profile in children before a change to 3-month regimen
	2. Parallel DOTs for children on preventive treatment with DOTs for index case	Some evidence that DOTs for preventive treatment would be effective [8]	Increased cost to provide DOTs, although economies of scale optimised if in parallel to index case DOTs	Introduce a modified DOTs programme for preventive treatment in parallel and overlapping with index case DOTs. Before and after evaluation.

DOT, directly observed therapy; INH, Isoniazid; RIF, rifampicin.

### Gap Analysis

During the gap analysis, disparities between current and ideal practices are quantified for each component. Table 2 shows examples of how each gap can be identified and described. This is undertaken for each component of the situational analysis. In most situations, it is expected that the extent of the gap can be clearly defined. However, it may be necessary to estimate the gap based on limited information. If this estimate is too imprecise, further research may be required to define the gap.

### Options Analysis

Following the identification of gaps between the current and ideal practices, options for closing these are reviewed. Each option is considered for scientific evidence of efficacy, feasibility in the specific setting, and cost evaluation from case contact household and societal perspectives. For each gap, an option to take forward for implementation is then recommended. Table 3 shows how the options can be presented.

Besides indicating the best possible intervention to improve the program, the options analysis will also lead to identification of information gaps in the literature where options for addressing an aspect of the current situation are unclear or unproven. In such circumstances, research may need to be undertaken to provide the necessary information. These research projects may range from simple cross-sectional evaluations to randomised intervention trials with follow-up. However, it is anticipated that a “best guess” for a decision on a way forward in some areas may simply be pragmatically necessary to develop a multi-component management plan that can be piloted in reasonable time.

### Clinical Evaluation of Child Contacts with Symptoms of TB Disease

A child TB contact management plan with a view to routine use of preventive treatment in those without symptoms of disease needs to include an approach to the diagnosis/exclusion of TB disease in symptomatic contacts of any age. The implication of using the WHO symptom-based approach is that those children who are asymptomatic can go directly onto preventive treatment, while those with defined symptoms immediately enter a clinical evaluation plan that either leads to full TB treatment for disease or, if active disease is excluded, to commencement of preventive treatment (see Box 1). Indeed, the process of contact screening will improve case-detection and treatment of TB because of the high prevalence of disease in contacts of any age [20]. The International Union Against Tuberculosis and Lung Disease has developed training tools that include management algorithms for the evaluation of symptomatic children [21]. It is recommended that each NTP develop a clinical evaluation plan using the following steps:

1. Design and implementation of a clinical management algorithm following input from paediatric services at the provincial level.
2. A clear decision pathway leading to anti-TB treatment or preventive treatment.
3. A follow-up plan.
4. Evaluation of the overall strategy.

### Implementation and Evaluation

From the options analysis, a comprehensive strategy is then developed for implementation. This can be piloted and rolled out after modification. After implementation, a monitoring and evaluation strategy is put in place using measurable indicators and key research tools as described in Table 1. A before and after indicator approach is well suited to multi-component public health intervention in TB control [22], although more complex implementation research tools could be used [23]. It is anticipated that such an “effectiveness study” would be conducted over approximately 2 years. Longer-term effectiveness evaluation could be incorporated through various methods. For example, the same studies could be repeated, or there may be a focus on under-performing areas with other areas subject to less intensive study.

### Operational Considerations

Operational research is, by definition, intimately related to the operations of the NTP. Therefore, the implementation plan in particular requires project management and funding to be coordinated between operations managers and operational researchers. Discussions should be held at an early stage regarding the coordination of changes to operational activities and the accompanying operational research. One solution is to establish a steering committee of key people [24]. This group needs to be carefully constructed, with expertise in operations and operational research, and have clear accountabilities and terms of reference. A key task of this steering group is to ensure that the new strategy is consistent with and incorporated into the National Guidelines, that it is successfully rolled out across the country, and is evaluable and sustainable.

## Conclusions

There is a significant gap between policy and practice in relation to child contacts of TB cases across the developing world. Here, we have proposed a way forward to address this problem, combining a HNA framework with research tools and a workable approach to clinical evaluation. We envisage that this approach will help close the gap between policy and practice in TB control in children. As such, it will contribute to the achievement of Millennium Development Goal number four, to reduce child mortality. In addition, it will help reduce the reservoir of *M. tuberculosis* in the community, which is necessary for TB elimination. Since this is an operations and operational research plan it will need to be carefully coordinated with local NTP activities, and a steering committee is strongly advised to facilitate this. A limited number of urban and rural sites per country need to adopt the approach described here, as resulting new routine practice can be rolled out more widely. Evidence from the application of the approach in different settings will also be able to inform the options analyses of others. Ultimately, the solutions that are identified and implemented on the basis of this approach will need to be sustainable. A key component will be building the capacity of local staff in operational research. Proper integration of operational research into NTP activities will only enhance global TB control.

## Abbreviations

HNA: health needs assessment
NTP: National TB Control Program
TB: tuberculosis
WHO: World Health Organization
